# Incidental findings in female pelvis MRI performed for gynaecological malignancies

**DOI:** 10.1186/s13244-025-02006-5

**Published:** 2025-06-27

**Authors:** Silvia Bottazzi, Roberta V. Ninkova, Luca Russo, Andrea Ponsiglione, Benedetta Gui, Daniela Demundo, Massimo Imbriaco, Aradhana M. Venkatesan, Evis Sala, Stephanie Nougaret, Lucia Manganaro, Stefania Rizzo

**Affiliations:** 1 Dipartimento Diagnostica per Immagini e Radioterapia Oncologica, Fondazione Policlinico Universitario A. Gemelli IRCCS, Rome, Italy; 2 Department of Radiological Sciences, Oncology and Pathology, Sapienza University of Rome, Rome, Italy; 3 Department of Experimental Medicine, Sapienza University of Rome, Rome, Italy; 4 Dipartimento Universitario di Scienze Radiologiche ed Ematologiche, Università Cattolica del Sacro Cuore, Rome, Italy; 5 Department of Advanced Biomedical Sciences, University of Naples “Federico II”, Naples, Italy; 6 Imaging Institute of Southern Switzerland, Ente Ospedaliero Cantonale (EOC), Lugano, Switzerland; 7 Division of Diagnostic Imaging, Department of Abdominal Imaging, The University of Texas MD Anderson Cancer Center, Houston, TX USA; 8 INSERM, PINKCC lab, Montpellier Cancer Research Institute, University of Montpellier, Montpellier, France; 9 Department of Radiology, University of Montpellier, Montpellier, France; 10 Faculty of Biomedical Sciences, Università della Svizzera Italiana (USI), Lugano, Switzerland

**Keywords:** Incidental findings, Magnetic resonance imaging, Pelvis, Gynaecological imaging

## Abstract

**Abstract:**

Incidental findings on female pelvic MRI present diagnostic challenges and may have significant clinical implications. Defined as abnormalities unrelated to the primary imaging indication, these findings have become increasingly prevalent with the expanded use of MRI in gynaecological practice. Standard gynaecological MRI protocols, incorporating T1- and T2-weighted sequences, diffusion-weighted imaging, and contrast-enhanced sequences, facilitate the characterisation of numerous extra-gynaecological abnormalities, ranging from benign to critical lesions. This review proposes a compartment-based approach for identifying extra-gynaecological findings, discussing their imaging characteristics and differential diagnoses. This approach may help radiologists systematically assess incidental findings, potentially improving the recognition of clinically relevant abnormalities and supporting timely clinical decision-making.

**Critical relevance statement:**

Incidental extra-gynaecological findings on pelvic MRI can present significant diagnostic challenges. Systematic evaluation of incidental extra-gynaecological findings on pelvic MRI can improve radiologists’ awareness of clinically relevant abnormalities.

**Key Points:**

- Extra-gynaecological incidental findings on pelvic MRI are common and range from benign to malignant conditions.
- A compartment-based classification—dividing the female pelvis into anterior, lateral, posterior, musculoskeletal, and miscellaneous compartments—provides a systematic framework for interpretation.
- Thorough assessment of all MRI sequences, including large field-of-view images, may help identify clinically relevant incidental findings.

**Graphical Abstract:**

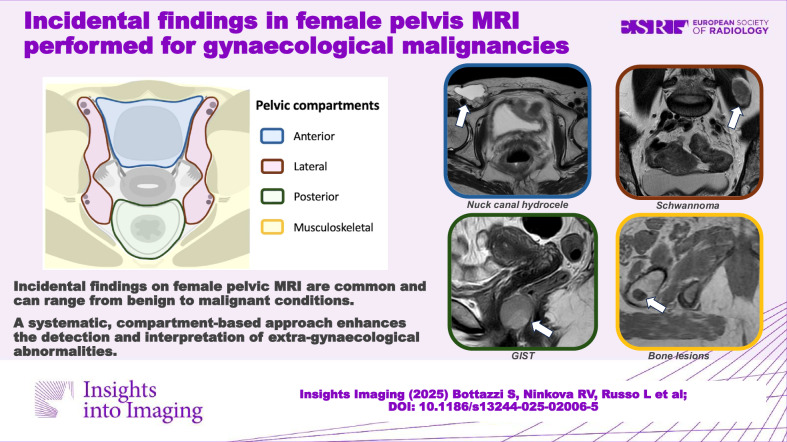

## Introduction

An incidental finding refers to an abnormality detected during an imaging study conducted for an unrelated purpose [1, 2].

In the last decades, the broader availability of magnetic resonance imaging (MRI) has led to an exponential increase in the number of pelvic imaging studies performed for gynaecological reasons. MRI is currently recommended for staging cervical cancer, for the pre-operative evaluation of endometrial and vulvar cancer, for characterisation of ultrasound-indeterminate adnexal masses, as well as for the assessment of deep endometriosis and atypical myometrial masses and in the recurrence setting [3–9]. In line with European Society of Urogenital Radiology (ESUR) guidelines, all MRI protocols for the aforementioned clinical scenarios include large field-of-view (FOV) T1- and/or T2-weighted images (WI), diffusion-weighted images (DWI) and, sometimes, post-contrast images [10–15]. Consequently, numerous extra-gynaecological findings can be encountered and may pose significant diagnostic challenges for the radiologist (Table 1).

**Table 1:** Incidental findings in female pelvis MRI according to compartments

Compartment	Incidental finding	
	Benign	Malignant
Anterior	Urinary stones, urachal cyst, urethral diverticulum (UD), Nuck cyst	Urachal carcinoma, bladder cancer
Lateral	Schwannoma, intravenous leiomyomatosis, appendiceal mucocele and mucinous neoplasms, pelvic congestion syndrome	Mucinous neoplasms
Posterior	Peritoneal inclusion cyst, Tailgut cyst, colonic diverticulosis and diverticulitis, perianal abscess, gastrointestinal stromal tumours (GISTs), extramedullary haematopoiesis	Colorectal cancer, GISTs
Musculoskeletal	Incidental bone lesions, sacroiliitis, stress fracture, distension of the iliopsoas bursa, iliopsoas bursitis	Incidental bone lesions
Miscellanea	Pelvic splenosis, epiploic appendagitis, mesenchymal tumours	Mesenchymal tumours

The prevalence of incidental findings on pelvic MRI varies significantly across studies, ranging from 42% to 98% [16–19]. Their clinical relevance ranges from common benign abnormalities (e.g. degenerative changes) to highly critical discoveries, including unexpected cancerous lesions [16, 20].

**Fig. 1:**
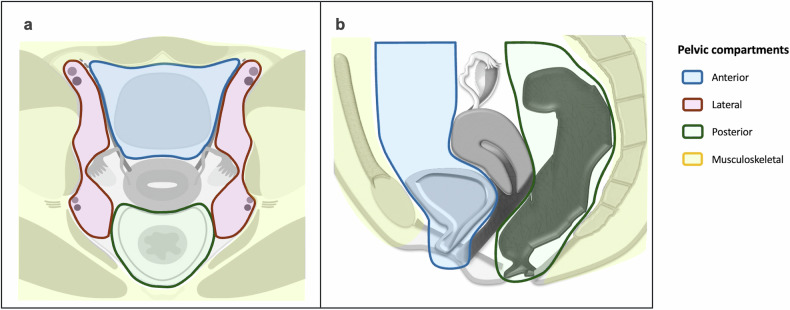
Illustration of the pelvic compartments in the axial (**a**) and sagittal planes (**b**)

This review outlines various incidental findings potentially encountered in daily practice, organised in a compartmentalised format to provide practical guidance for radiologists interpreting gynaecological MRI [21, 22]. Focusing on extra-gynaecological findings, the female pelvis has been divided into anterior, lateral, posterior, musculoskeletal and miscellaneous (any) compartments, using the reproductive system as a central reference (Fig. 1).

### Anterior compartment

#### Urinary stones

**Fig. 2:**
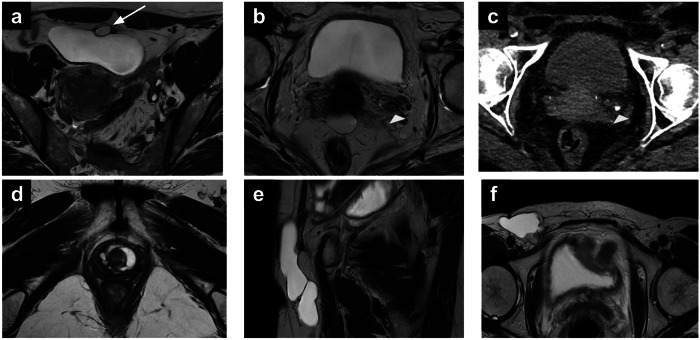
Urachal cyst (**a**). Axial T2-WI (**a**) showing a urachal cyst (arrow) in a 43-year-old patient undergoing pelvic MRI for endometriosis. Ureteric calculus (**b**, **c**). Axial T2-WI (**b**) of a 69-year-old patient with recurrent vulvar cancer showing a filling defect in the left distal ureter (arrowhead), with associated ureteric wall thickening, then confirmed as a calcified calculus at subsequent CT. UD (**d**). Axial T2-WI (**d**) of a 53-year-old patient with locally advanced cervical cancer showing a multicystic lesion with fluid content surrounding the urethra, in keeping with a UD. Compression of the anterior vaginal wall can be noted. Nuck canal hydrocele (**e**, **f**). Sagittal T2-WI (**e**) and axial T2-WI (**f**) of a 54-year-old patient with vaginal cancer; an elongated cystic lesion with fluid content and thin walls can be noted in the right inguinal region (canal of Nuck), representing a Nuck canal hydrocele. WI, weighted images

Urinary stones affect up to 12% of the population, predominantly males. Patients are usually symptomatic, experiencing severe pain migrating from the flank region to the lower back, pelvis and genital region. Associated symptoms may include nausea, vomiting, fever and haematuria. The detection is usually achieved through a CT scan [23, 24]. As an incidental finding on MRI, urinary calculi appear as signal voids on T1-WI and T2-WI, or as filling defects in a dilated ureter (Fig. 2) [25, 26]. Ureteric wall thickening with associated contrast enhancement and surrounding fat stranding may be observed in the case of associated inflammation [27].

#### Urachal cyst

During embryonic life, the urachus connects the foetal bladder to the allantois. Normally, it obliterates before birth, forming the median umbilical ligament, which connects the bladder to the umbilicus. Urachal abnormalities occur when this structure persists after birth. They have an approximate incidence of 1 in 5000 with a slight male predominance [28, 29].

Urachal cysts (UC) represent 30% of urachal abnormalities. They are usually detected incidentally on imaging in asymptomatic patients but can rarely cause abdominal pain due to haemorrhage or rupture. On MRI, UC appear as round midline lesions, with thin walls and fluid content, showing high signal intensity (SI) on T2-WI and low SI on T1-WI, sometimes containing calcifications (Fig. 2). An associated umbilical-urachal sinus, which represents dilatation of the urachus umbilical end, can be found [28–30].

Prophylactic excision of UC in asymptomatic adult patients, though controversial, can be considered to prevent infections or malignant transformation [29, 30].

#### Urethral diverticulum (UD)

UD represents a focal dilatation of the urethra, most commonly seen in women (0.6–6%). Congenital female UD is rare. Patients can be asymptomatic or present with urinary symptoms [31, 32].

UD typically originates from the posterolateral wall of the middle third of the urethra [33]. On sagittal T2WI, these lesions can elevate the bladder base, mimicking the shape of an enlarged male prostate [31, 34, 35].

On MRI, UD appears as a fluid-filled cavity, usually located anterior to the vagina, showing high SI on T2-WI (Fig. 2). Contrast administration can help detect complications such as infection or malignant transformation. In cases of infection, UD may demonstrate thickened walls, multiple enhancing septa and heterogenous endoluminal material. Cancers arising in the UD are extremely rare, with fewer than 100 cases described [31, 34, 36]. Asymptomatic patients can be managed conservatively with follow-up, whereas surgery is usually the primary treatment option in cases of symptomatic UD [31, 37].

#### Nuck cyst

The canal of Nuck (CN) is an extension of the parietal peritoneum that follows the inguinal canal alongside the round ligament of the uterus, extending into the labia majora. Normally, the CN undergoes complete obliteration within the first year of life. However, when the canal remains patent, abnormalities can arise. The most common abnormality in adults is the encysted hydrocele, which results from partial closure of the CN at its proximal and distal ends, leaving a patent central portion.

On MRI, Nuck cysts appear as fluid-filled structures with thin walls and variable shape (round, elongated, comma-shaped, cyst-in-cyst appearance) showing high SI on T2-WI (Fig. 2) [38, 39].

Surgical excision with ligation of the processus vaginalis neck at the deep inguinal ring represents the treatment of choice for symptomatic Nuck cysts, while conservative treatment may be an option for asymptomatic patients.

#### Urachal carcinoma

Malignant urachal carcinomas are rare, with an incidence of 0.18 per 100,000 individuals annually. They represent approximately 0.2% of bladder cancers, with men being affected twice as often as women [28, 29, 40]. Adenocarcinoma accounts for more than 80% of these tumours [30, 40].

Urachal carcinomas originate from urachal remnants and are typically located in the juxtavesical portion of the urachus. These lesions tend to invade the bladder dome but exhibit more prominent extravesical growth compared to non-urachal bladder cancers [30, 41]. Urachal carcinoma is usually clinically silent in early stages; when symptomatic, haematuria—due to bladder wall invasion—and a palpable pelvic mass are the most common clinical findings [30, 41].

On MRI, urachal carcinomas appear as large, heterogeneous masses, with irregular margins, and an avidly enhancing solid component. Areas of high SI on T2-WI can be found due to mucin content. Peripheral calcifications, found in up to 70% of cases, are considered pathognomonic [30, 41].

#### Bladder cancer (BC)

BC is the 10th most diagnosed neoplasm worldwide. It predominantly affects men, with a male-to-female ratio of 3–4:1. The incidence increases with age, with most cases occurring after the age of 70 [42–44]. Urothelial carcinoma accounts for up to 90% of BC cases [45]. Asymptomatic macrohaematuria is the most common symptom, although patients may also present with microhaematuria, irritative urinary symptoms or obstructive urinary symptoms, in case of advanced disease.

Lesions can present as flat, papillary or pedunculated, with an intramural or exophytic growth or with no mass effect [46, 47].

On MRI, BC appears as an area of intermediate SI on T2-WI, showing restricted diffusion on DWI and early enhancement following contrast administration. Disruption of the hypointense line on T2WI, representing the muscular layer, is indicative of muscle-invasive BC. DWI images are useful to spot small lesions and to evaluate the presence of muscle invasion, while post-contrast images are crucial to differentiate BC from non-malignant findings such as clots or debris [43, 47–49].

The Vesical Imaging Reporting and Data System classification is a useful tool, that has demonstrated high sensitivity and specificity for detecting muscle-invasive BC, an adverse prognostic factor [46, 50–55].

### Lateral compartment

#### Schwannoma

**Fig. 3:**
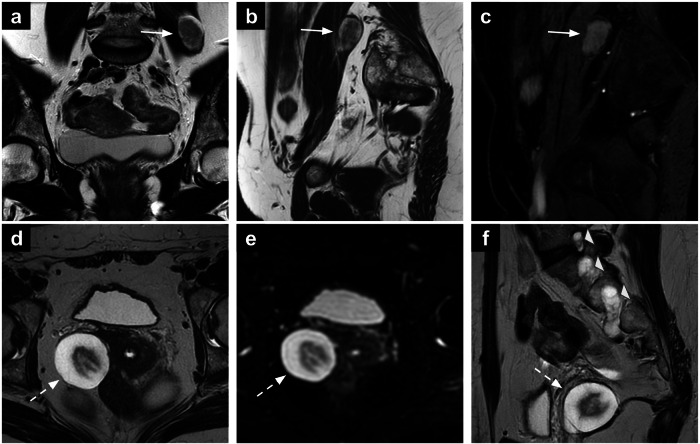
Incidental finding of intramuscular schwannoma in a patient with suspected endometriosis (**a**–**c**). A well-circumscribed mass (arrow) showing intermediate-to-high SI on T2-WI is seen in the left psoas muscle (**a**, **b**). On post-contrast T1-WI, the lesion shows homogeneous enhancement (**c**). Cystic degeneration of a schwannoma in a 44-year-old patient (**d**–**f**). The lesion (**d**–**f**) demonstrates high SI on T2-WI (**d**) and low *b*-value DWI (**e**). On sagittal T2-WI (**f**), there is evidence for multiple lesions with similar signal characteristics associated with the exiting sacral nerve roots (arrowheads). WI, weighted images

Macroscopically, schwannomas are generally well-encapsulated, and smaller tumours are typically solid. Calcifications are present in approximately 23% of cases, while cystic degeneration is observed in nearly 60% [56]. On MRI, schwannomas typically appear as well-circumscribed masses displacing, but not infiltrating, adjacent structures. They exhibit low SI on T1-WI, high SI on T2-WI, and heterogeneous enhancement on post-contrast T1-WI. In larger lesions, features such as cystic changes, haemorrhage, and fatty degeneration may be observed, while calcifications are rare (Fig. 3) [57, 58]. Accurate identification is essential to avoid unnecessary surgical interventions, as many cases can be managed conservatively.

#### Intravenous leiomyomatosis

Intravenous leiomyomatosis is a rare benign smooth muscle tumour arising either from the wall of a vessel or from a uterine leiomyoma. The aetiology remains uncertain. The condition primarily affects women of reproductive or perimenopausal age, with a median age of 45 years, and is commonly observed in individuals with a history of uterine leiomyomas or prior hysterectomy [59, 60]. While histologically benign, intravenous leiomyomatosis exhibits a growth pattern characterised by intraluminal proliferation within venous structures. Typically, these tumours grow slowly and have a favourable prognosis. Patients may be asymptomatic or present with symptoms related to uterine leiomyomas. In advanced cases, the tumour may extend into the iliac veins, inferior vena cava, and even the right atrium or pulmonary artery, potentially resulting in life-threatening complications [61].

**Fig. 4:**
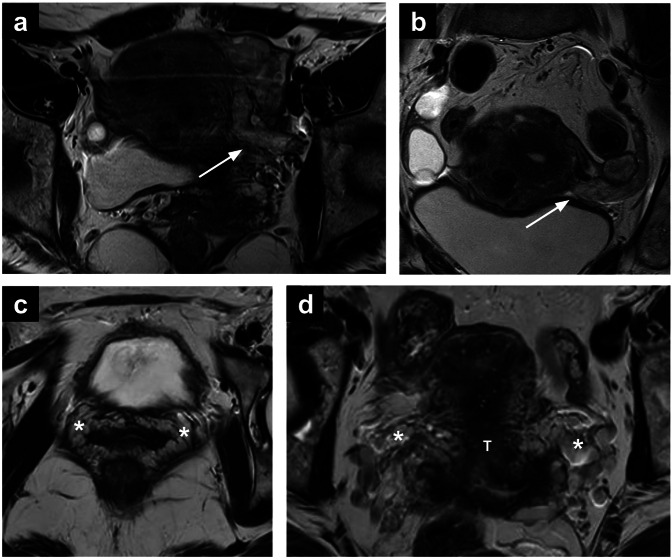
Intravascular leiomyomatosis (**a**, **b**). Axial and coronal T2-WI (**a**, **b**) showing incidental finding of left intravascular leiomyomatosis (arrow) in a 44-year-old patient who underwent MRI for characterisation of multiple uterine leiomyomas. The lesion has an elongated shape and intermediate-to-high SI on T2-WI. Pelvic congestion (**c**, **d**). Axial and oblique coronal T2-WI (**c**, **d**) showing bilateral pelvic congestion in a 46-year-old patient undergoing MRI for cervical cancer (T) staging. If parametrial invasion is absent and vessel enlargement is symmetric (like in this case), pelvic congestion is the most likely diagnosis. SI, signal intensity; WI, weighted images

MRI can play a key role in diagnosing IVL and assessing its extent. The tumour typically appears as an isointense mass on T1-WI with heterogeneous high SI on T2-WI, and marked enhancement on post-contrast T1-WI (Fig. 4). Filling defects are commonly observed in parauterine vessels and the inferior vena cava [62].

Surgical excision is the treatment of choice and often requires a multidisciplinary approach, especially in cases involving extensive venous or cardiac structures.

When the inferior vena cava is involved, the differential diagnosis should include renal cell carcinoma, with associated tumour thrombus [62].

#### Appendiceal mucocele and appendiceal mucinous neoplasm

Appendiceal mucocele is a rare condition characterised by abnormal distention of the appendix due to luminal obstruction, from either neoplastic or non-neoplastic causes. Non-neoplastic aetiologies include mucosal hyperplasia and simple retention cysts, which may arise from factors such as obstructing appendicoliths, endometriosis, external compression or inflammatory processes. Primary appendiceal neoplasms are uncommon, accounting for about 1% of appendectomy specimens. Appendiceal mucocele of any origin is even rarer, with an incidence of 0.2–0.3% [63, 64]. The condition shows a female predominance and is most commonly diagnosed in individuals aged 50–60 years [63–65].

**Fig. 5:**
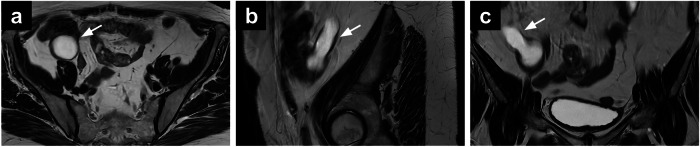
Axial (**a**), sagittal (**b**) and coronal (**c**) T2-WI in a 66-year-old patient on follow-up for cervical adenocarcinoma showing a cyst-like mass (arrows) in the right iliac fossa region, close to the caecum, suggestive of appendiceal mucocele. WI, weighted images

On MRI, a mucocele typically appears as a well-circumscribed, tubular cystic structure in the right lower quadrant with high SI on T2-WI (Fig. 5). On T1-WI, it exhibits low to intermediate SI, depending on the mucin content and viscosity. In mucin-rich lesions, a stratified or layered appearance, referred to as the “onion skin” sign, may be observed [66]. Post-contrast sequences often reveal mild to moderate wall enhancement, raising suspicion for neoplastic transformation [66]. DWI provides additional diagnostic value, as restricted diffusion is more commonly seen in malignant lesions. MRI plays a pivotal role not only in characterising the mucocele but also in assessing associated complications such as rupture, peritoneal dissemination, or secondary pseudomyxoma peritonei, crucial for guiding surgical management.

#### Pelvic congestion syndrome (PCS)

PCS is a common condition characterised by chronic pelvic pain persisting for more than six months, in the absence of inflammatory disease. It is frequently associated with venous insufficiency and varicosities within the pelvic region [67].

The prevalence of PCS among patients presenting with chronic pelvic pain ranges from 12% to 33% [68]. PCS predominantly affects premenopausal, multiparous women, who often report chronic pelvic pain accompanied by dysmenorrhoea. These symptoms are typically exacerbated by prolonged standing or during and after sexual intercourse [69].

Characteristic findings on MRI include tortuous pelvic veins with increased calibre (typically exceeding 5 mm in diameter) and slow-flow phenomena, which are evident on T2-WI and contrast-enhanced sequences (Fig. 4). Digital Subtraction Angiography (DSA) remains the gold standard for diagnosing pelvic venous disease, providing high-resolution, real-time visualisation of pelvic venous pathology. It enables precise assessment of venous reflux, dilation, and collateral circulation, playing a key role in both diagnosis and guiding endovascular interventions such as ovarian vein embolisation. However, as an invasive procedure associated with radiation exposure and contrast-related risks, DSA is primarily reserved for patients requiring intervention or those with inconclusive non-invasive imaging findings [70, 71].

### Posterior compartment

#### Peritoneal inclusion cyst

**Fig. 6:**
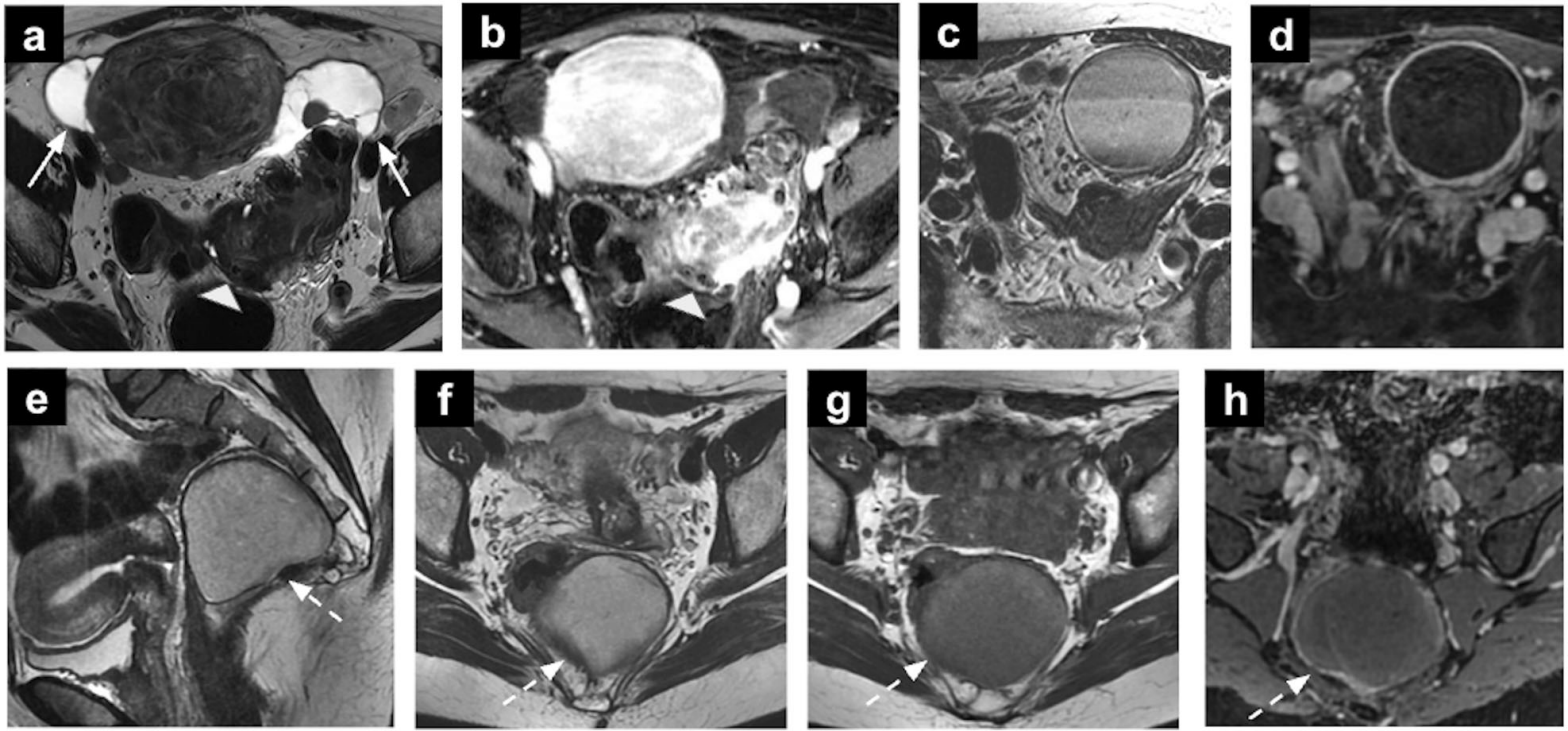
Peritoneal inclusion cysts and diverticulitis (**a**, **b**). Axial T2-WI (**a**) and post-contrast T1-WI (**b**) showing inclusion cysts (arrows) in a 63-year-old patient undergoing MRI for para-ovarian cysts characterisation. They appear as thin-walled and fluid-filled structures without contrast enhancement, conform to the surrounding uterus and ovaries, due to their reactive nature to peritoneal adhesions. Differential considerations include mesenteric cysts (**c**, **d**), which, in contrast, are typically isolated and do not conform to adjacent structures. In this case of peritoneal inclusion cysts, there is also evidence of diffuse sigmoid wall thickening (arrowhead in **a**) with avid enhancement (arrowhead in **b**), in keeping with concomitant diverticulitis. Tailgut cyst (**e**–**g**). Sagittal and axial T2-WI (**e**, **f**), axial T1-WI (**g**) and post-contrast T1-WI (**h**) showing a tailgut cyst (dotted arrow). It appears as a retrorectal thin-walled unilocular cystic lesion with high-SI and intermediate-to-low-SI content on T2-WI (**e**, **f**) and T1-WI (**g**), respectively. No enhancing component is noted. SI, signal intensity; WI, weighted images

Peritoneal cysts are rare, benign lesions arising from the mesothelium of the peritoneum. Peritoneal cysts are usually asymptomatic but may occasionally cause symptoms such as abdominal discomfort or pelvic pain when they reach a significant size. These cysts are often located in the peritoneal cavity and may be seen in association with adhesions or prior abdominal surgeries. Macroscopically, they typically appear as well-defined, thin-walled, fluid-filled structures with homogenous high SI on T2-WI and low SI on T1-WI (Fig. 6). They do not enhance on post-contrast sequences, consistent with their benign, non-vascular nature. MRI is valuable in differentiating peritoneal cysts from other cystic pelvic lesions [72], including mesenteric cysts, which originate within the mesentery and are closely associated with intestinal structures [73].

Follow-up or intervention is generally unnecessary unless there is diagnostic uncertainty or clinical suspicion of complications such as infection or haemorrhage.

#### Tailgut cyst

Tailgut cysts, also known as retro-rectal cystic hamartomas, are rare congenital lesions arising from remnants of the embryonic hindgut. The tailgut, represents the distal portion of the embryonic hindgut located below the developing anus. Normally, the tailgut regresses by the eighth week of embryonic development; however, incomplete regression can result in the formation of a tailgut cyst [74, 75].

Tailgut cysts are more frequently observed in women and are commonly diagnosed during middle age (30–60 years), although they can occur at any age. They are typically asymptomatic and incidentally detected. When symptomatic, patients may present with nonspecific complaints such as abdominal pain, constipation, or pelvic discomfort. Rarely, tailgut cysts can undergo malignant transformation.

Macroscopically, tailgut cysts appear as unilocular or multilocular, thin-walled structures filled with mucoid material. They are usually located in the retrorectal or presacral space and measure several centimetres in diameter. On MRI, these cysts typically exhibit high SI on T2-WI and variable SI on T1-WI, depending on the presence of mucin, proteinaceous material, or haemorrhage within the cyst (figure although typically benign, cysts are frequently managed with surgical excision, even in asymptomatic cases, due to the potential risk of complications, such as infection or inflammation, which may result in cyst wall fibrosis and degradation of the epithelial lining [76].

The differential diagnosis for presacral cystic masses includes several entities, such as epidermoid cysts, dermoid cysts, rectal duplication cysts, anal gland cysts, cystic lymphangiomas, and anterior meningoceles [77].

#### Colonic diverticulosis and diverticulitis

Diverticular disease is characterised by the presence of multiple outpouchings, most commonly located in the sigmoid colon or left colon. It predominantly affects older individuals, with approximately 80% exhibiting the condition by the age of 80 [78]. However, diverticular disease is estimated to affect 5–10% of individuals by the age of 45. Symptoms of diverticulitis typically include persistent pain and tenderness in the left iliac fossa.

Diverticulitis is characterised by bowel wall thickening, pericolonic stranding, and the presence of diverticula (Fig. 6). T2-WI is useful in identifying the inflamed intestinal wall and detecting associated intraperitoneal fluid. Intravenous gadolinium-enhanced sequences further aid in identifying inflammation and abscess formation.

Complicated diverticulitis may present with phlegmon, small abscess, perforation, or fistulas, which occur in approximately 14% of cases. The most common type of fistula is the enterovesical fistula, identifiable on MRI by the presence of air within the bladder.

Treatment is guided by disease stage and patient comorbidities, with conservative management for localised disease, percutaneous drainage for large abscesses, and emergency surgery for advanced stages.

The differential diagnosis primarily includes colorectal cancer (CRC) and inflammatory bowel disease (IBD) [79, 80].

#### CRC

CRC is the third most prevalent malignancy in women [44, 81, 82]. Risk factors for CRC include familial and personal medical history, lifestyle factors, and genetic predispositions. Early-stage CRC may be asymptomatic, or patients may present nonspecific symptoms such as rectal bleeding, abdominal pain, altered bowel habits, fatigue and unexplained weight loss [83]. On MRI, CRC commonly manifests as irregular bowel wall thickening with intermediate SI on T2-WI, demonstrating restricted diffusion on high *b*-value DWI and low values on apparent diffusion coefficient (ADC) map [84]. The identification of lymphadenopathy and extramural extension, including invasion of adjacent structures, is critical for staging and treatment planning. Definitive diagnosis requires recto-colonoscopy with histopathological confirmation of malignancy.

#### Perianal abscess

**Fig. 7:**
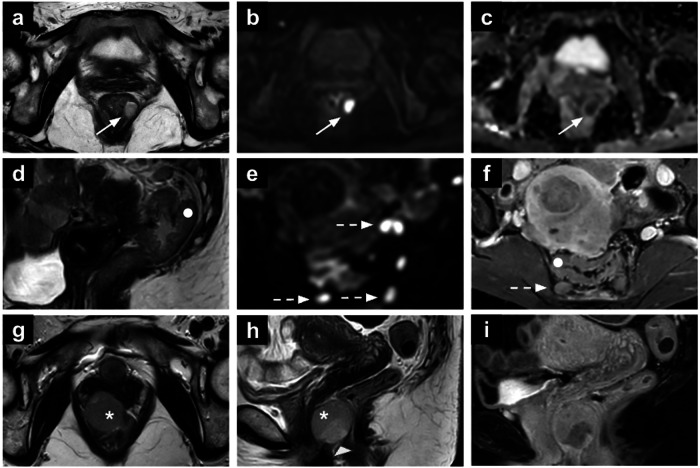
Rectal abscess (**a**–**c**). Axial T2-WI (**a**), high *b*-value DWI (**b**) and the ADC map (**c**) of a 45-year-old patient underwent MRI for endometrial cancer staging revealing an intersphincteric abscess in the upper third of the left anal canal (arrows). Ulcerative colitis (**d**–**f**). Sagittal T2-WI (**d**), axial DWI (**e**) and post-contrast T1-WI (**f**) showing incidental ulcerative colitis, characterised by diffuse rectal wall thickening with increased enhancement (white dot in **d**–**f**). Multiple reactive mesorectal nodes are also noted (dotted arrows in **e**, **f**). GIST (**g**–**i**). Axial T2-WI (**g**), sagittal T2-WI (**h**) and post-contrast T1-WI (**i**) in a 51-year-old patient undergoing MRI for adnexal mass characterisation. The images show a round-shaped mass in the recto-vaginal septum (arrowhead), displacing the vagina anteriorly and the rectum posteriorly. Two components can be recognised: a solid component (asterisk) showing intermediate SI on T2-WI, and a necrotic component (arrowhead in h) showing high SI on T2-WI and lack of enhancement. SI, signal intensity; WI, weighted images

Perianal abscesses are infective-inflammatory collections that can arise from anal gland obstruction or can be associated with chronic inflammatory conditions [85]. On MRI, abscesses appear hyperintense on T2-WI due to their fluid content, with chronic abscesses showing hypointensity on both T1-WI and T2-WI sequences (Fig. 7). Moreover, the contrast-enhanced imaging aids in identifying abscesses with associated fistulas, differentiating between acute and healing phases based on contrast enhancement patterns. Vivid wall enhancement is noted during the acute phase of an abscess, while progressively homogeneous and late enhancement is characteristic of the healing phases. Additionally, DWI may offer enhanced sensitivity in detecting fistulas, serving as an alternative to post-contrast imaging for further evaluation. Early identification is critical, as it facilitates timely surgical intervention, including drainage, and helps prevent complications like fistula formation [86, 87].

#### Gastrointestinal stromal tumours (GISTs)

GISTs are rare mesenchymal neoplasms that arise from the interstitial cells of Cajal within the gastrointestinal tract, most commonly in the stomach and mid-distal small intestine (5%). They are commonly diagnosed in the middle-aged and elderly population [88, 89]. Macroscopically, these tumours can present as well-circumscribed soft tissue masses with heterogeneous SI, depending on the presence of necrosis, haemorrhage, or cystic degeneration. It typically presents as a mass with exophytic or intraluminal growth [90].

On MRI, GISTs often exhibit hyperintense signal on T2-WI, while they may reveal variable SI based on the internal tumour composition on T1-WI. Post-contrast sequences typically demonstrate avid enhancement of the solid components.

Although GISTs are primarily diagnosed through endoscopic or surgical evaluation, pelvic MRI can reveal extraluminal manifestations or associated complications, such as mass effect on adjacent structures or intraperitoneal dissemination in advanced cases (Fig. 7). The incidental detection of a GIST on pelvic MRI highlights the importance of evaluating all abdominal and pelvic structures during imaging, particularly in asymptomatic patients or those with vague abdominal symptoms. En bloc surgical resection represents the primary treatment modality for GISTs, with or without adjuvant chemotherapy [91].

The main differential diagnoses include gastrointestinal lymphoma, leiomyoma, and carcinoid tumours [92].

#### Extramedullary haematopoiesis (EMH)

EMH is a rare condition in which haematopoietic tissue develops outside the bone marrow, often as a compensatory response to chronic anaemia, bone marrow dysfunction, or haematologic disorders such as thalassaemia or myelofibrosis.

The most common sites of EMH are the liver and the spleen, as the remnants of foetal blood production [93]. Less than 5% of EMH cases occur outside of these two organs, including the thoracic vertebral column, lymph nodes, visceral surfaces, lung, and in the presacral area [94, 95]. EMH can occasionally present in the pelvic region and may be incidentally identified on pelvic MRI.

**Fig. 8:**
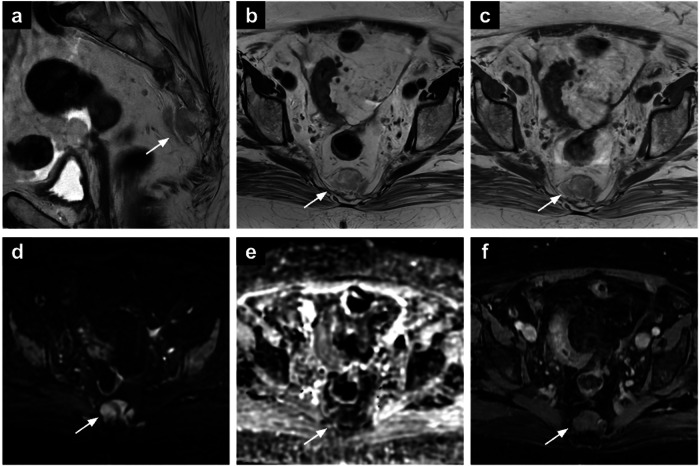
EMH in a 69-year-old patient who underwent hysterectomy and bilateral salpingo-oophorectomy for endometrial cancer. The lesion (arrows) shows intermediate-to-high SI on T2-WI (**a**, **b**) and T1-WI (**c**), in keeping with co-existing fat and soft-tissue components, the latter showing diffusion restriction (**d**, **e**) and mild enhancement (**f**). SI, signal intensity; WI, weighted images

On MRI, EMH typically appears as small well-defined or lobulated soft tissue masses with intermediate to low SI on T1-WI and variable SI on T2-WI, depending on cellularity and fat content (Fig. 8). Post-contrast enhancement is often mild to moderate, and associated findings such as fat signal within the lesion or adjacent skeletal abnormalities, such as bone expansion or marrow signal changes, may support the diagnosis. These masses carry a high risk of bleeding during biopsy, particularly near vital structures such as the spinal cord, where fine needle aspiration is preferred to minimise the risk of spinal cord compression, while treatment options include radiotherapy, surgical excision, or multiple blood transfusions [96]. Although EMH is usually asymptomatic, its recognition is important to differentiate it from malignancies or other soft tissue masses, particularly in patients with underlying haematologic disorders.

### Musculoskeletal system

#### Incidental bone lesions

**Fig. 9:**
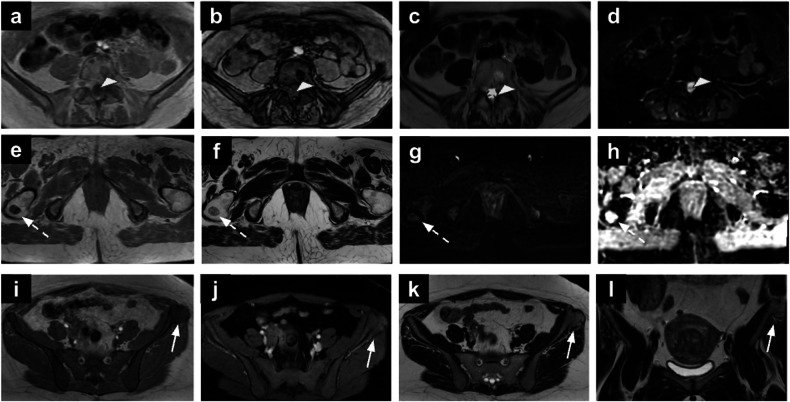
Incidental bone marrow lesions: haemangioma (**a**–**d**), enchondroma (**e**–**h**) and osteochondroma (**i**–**l**). Pelvic MRI (**a**–**d**) for cervical cancer staging in a 61-year-old patient showing a haemangioma in the L5 vertebral body. There is intralesional fat, depicted as signal drop between in-phase (**a**) and out-of-phase (**b**) images; the lesion (arrowheads) shows high SI on T2-WI (**c**) and moderate SI high *b*-value DWI (**d**). Incidental right femoral enchondroma (dotted arrows) in a 64-year-old patient: the lesion has well-defined borders, low SI on T1-WI (**e**) and intermediate-to-high SI on T2-WI (**f**), without significant diffusion restriction (**g**, **h**). Incidental left iliac osteochondroma (arrows) in a 48-year-old patient undergoing MRI for myometrial mass characterisation (**i**–**l**). The osteochondroma is characterised by the normally appearing bone marrow, continuous with the medullary cavity of the left iliac bone. SI, signal intensity; WI, weighted images

Incidental bone lesions have an overall frequency of up to 10% [97, 98]. In 2022, the Society of Skeletal Radiology developed the bone reporting and data system (Bone-RADS) for incidentally encountered solitary bone lesions on CT and MRI to aid radiologists in the diagnostic management of incidental bone lesions on CT and MRI [2]. It considers T1-WI the primary assessment sequence, with lesions resembling subcutaneous fat or showing > 20% signal drop between in-phase and out-of-phase T1 sequences generally considered benign (e.g. focal red marrow islands, benign lipomas, haemangiomas) (Fig. 9) [2, 98]. If SI differs, post-contrast imaging is crucial: central or nodular enhancement raises suspicion, while non-enhancing lesions require further evaluation. For lesions with low T1 signal, evaluation of T2-WI is necessary. A low T2 SI generally indicates benignity, as seen in bone islands, though osteoblastic metastases, particularly from breast cancer, should also be considered. Conversely, lesions with higher T2 SI warrant careful evaluation for malignancy. MRI features suggestive of malignancy include cortical involvement, the halo sign, central or nodular enhancement, aggressive periostitis, soft tissue extension and pathological fracture [2, 98]. Clinical history, lesion location, and complementary imaging such as radiographs or CT scans refine risk evaluation and management decisions [97, 98].

Although not included in the bone-RADS scoring system, DWI can play a crucial role in bone marrow metastases detection, thanks to its excellent sensitivity (91–93%) and specificity (87–94%) [99, 100].

Furthermore, the acquisition of T1-Dixon images would allow relative fat-fraction maps calculation, where bone metastases show very low fat content (< 20%) compared to fat-containing lesions and normal bone marrow [101].

#### Sacroiliitis

Sacroiliitis is an inflammation of the sacro-iliac joints, causing inflammatory back pain and commonly the first evidence of spondyloarthritis. It can result from various conditions, including IBD, ankylosing spondylitis, psoriatic and reactive arthritis [102].

**Fig. 10:**
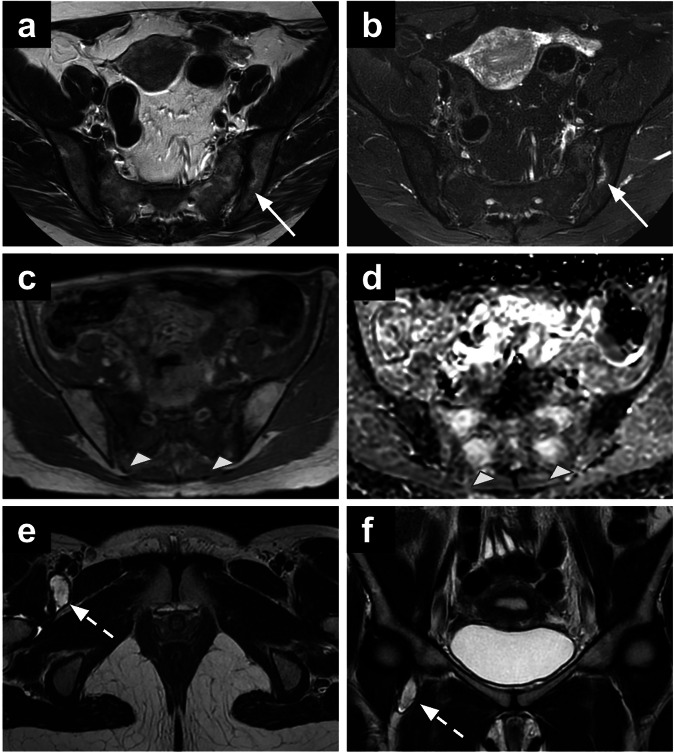
Sacroiliitis (**a**, **b**). Axial T2-WI (**a**) and STIR (**b**) images of a 50-year-old woman who underwent MRI for left ovarian lesion characterisation showing left active sacroiliitis (arrows). Pelvic insufficiency fracture (IF) (**c**, **d**). Bilateral thin obliquely oriented hypointense lines on axial T1-WI are noted in the sacrum of a 64-year-old patient on follow-up for cervical cancer treated with radiotherapy, with diffuse bone marrow oedema confirmed on ADC map, in keeping with IF. Synovial cyst (**e**, **f**). Axial (**e**) and coronal T2-WI (**f**) showing a synovial cyst arising from the right hip in a young woman with pelvic pain. STIR, short tau inversion recovery; WI, weighted images

On MRI, active sacroiliitis is characterised by the presence of subchondral bone marrow oedema, best visualised on T2-WI with fat saturation or short tau inversion recovery (STIR) images (Fig. 10) [103]. The symmetry vs laterality of the findings depends on the underlying cause [102].

Additional MRI findings indicative of sacroiliitis are erosive changes, which may appear as areas of high SI on T1-WI due to fat infiltration, ankylosis of the sacroiliac joints, joint space narrowing, and sclerosis predominant on the sacral side, especially in individuals under 40 years of age [98].

The identification of sacroiliitis on MRI should raise suspicion for an underlying spondyloarthropathy. However, correlation with clinical and laboratory findings is mandatory to avoid false positives, as several conditions can mimic sacroiliitis, including stress-related injury, osteoarthritis, among others [104].

#### Stress fracture

Stress fractures have an incidence of approximately 1% in the general population [105]. They are categorised into insufficiency and fatigue fractures, although, colloquially, the term “stress fracture” is used specifically to refer to fatigue fractures [105, 106].

Insufficiency fractures (IF) are due to a normal load on a weakened bone [106]. Common risk factors include osteoporosis and previous radiation therapy [102]. Post-pelvic radiotherapy fractures have an incidence ranging from 13% to 89%. These fractures are more common in middle-aged women [105, 107–109].

IFs are typically located in the sacrum, acetabula and pubis rami. They are usually bilateral and symmetrical. On MRI, they appear as ill-defined areas of oedematous changes in the bone marrow with contrast enhancement. In some cases, a hypointense fracture line is also visible. In the sacrum, this is typically parallel to the sacroiliac joint, often with a transverse component, creating an “H” appearance. The absence of diffusion restriction on DWI images in the fracture line helps the differential diagnosis with metastatic bone disease (Fig. 10) [102, 105, 110].

Conversely, fatigue fractures result from repetitive stress on a normal bone and are commonly seen in athletes, runners, dancers, etc. Typical locations include the medial and superolateral parts of the femoral neck, the sacrum and the pubic rami. On MRI, the presence of a hypointense fracture line on T1 and T2-WI is required to confirm the diagnosis of a stress fracture [102, 105, 106]. Generally, conservative management with rest and pain control is the preferred approach. However, some patients may not benefit from prolonged rest, making surgery a viable alternative. Therefore, a specialist orthopaedic evaluation is recommended to determine the most appropriate management strategy [111].

#### Distension of the iliopsoas bursa and iliopsoas bursitis

Cystic lesions around the hip are incidentally detected in up to 26% of patients. Synovial cysts are fluid-filled para-articular structures containing synovial fluid, functioning as a drainage reservoir for excessive joint fluid accumulation [112].

The iliopsoas bursa is a synovial sac located between the iliopsoas tendon and the anterior part of the hip, laterally to the femoral vessels. Inflammation, trauma, mechanical changes and other benign conditions can lead to the distension of the normally collapsed iliopsoas bursa [98, 102]. The terms “iliopsoas bursa distension” and “synovial cyst” are often used interchangeably due to their similar behaviour. On MRI, these lesions usually appear as fluid-filled, well-defined structures, with thin enhancing walls (Fig. 10). Communication with the adjacent hip joint is seen in up to 50% of cases. Inflammatory changes may present as thickening of the walls and intralesional debris. The presence of gas within the bursa is more likely due to vacuum phenomena rather than abscess formation; while the presence of an intralesional nodular enhancing component should be regarded as a suspicious finding [98, 110]. Correlation with clinical symptoms is necessary as iliopsoas bursitis is generally managed conservatively with activity modification, physical therapy and nonsteroidal anti-inflammatory drugs [113].

### Miscellanea (any compartment)

#### Pelvic splenosis

Splenosis is a benign condition characterised by the ectopic auto-transplantation of splenic tissue to different parts of the body. In 93% of cases, splenosis is caused by trauma and subsequent splenectomy [114]. The most common sites for splenic deposits include the left upper quadrant of the abdomen and pelvis. Typically, splenosis is asymptomatic and is discovered incidentally during imaging studies or surgical procedures performed for unrelated medical conditions [115].

**Fig. 11:**
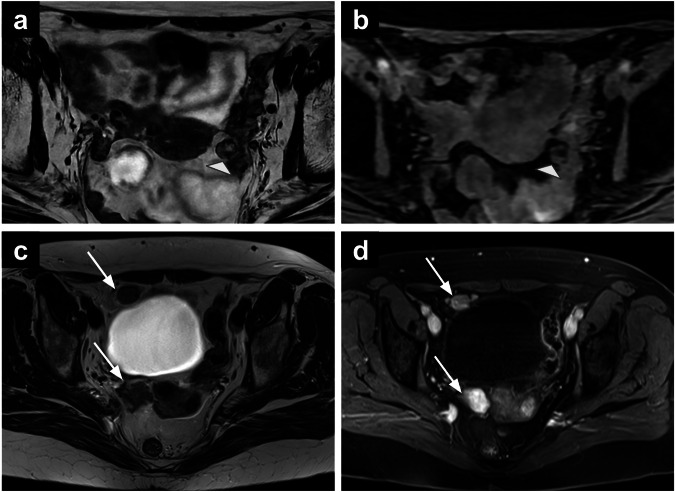
Appendigitis (**a**, **b**). Axial T2-WI (**a**) and fat-suppressed T1-WI (**b**) in a 48-year-old cervical cancer patient 3 months after CCRT showing epiploic appendagitis (arrowheads). Pelvic splenosis (**c**, **d**). Pelvic nodules (arrows in **c**, **d**) in a 59-year-old patient with breast cancer who underwent pelvic MRI for characterisation of para-uterine solid lesions detected on a transabdominal ultrasound. These lesions turned out to be sites of pelvic splenosis, related to a previous post-traumatic splenectomy. SI, signal intensity; WI, weighted images

The splenic nodules are often multiple and circular; their density, SI, and enhancement patterns generally resemble those of the splenic tissue (Fig. 11). Fine needle aspiration is the gold standard for diagnosing splenosis, while surgical excision and complete removal of pelvic splenosis nodules are not always required and should be guided by clinical symptoms [114].

Pelvic splenosis can mimic several clinical conditions, such as abdominal lymphoma, metastatic cancer, carcinomatosis, primary renal or hepatic malignancies, adenomas, endometriosis, or simple lymphadenopathy [115].

#### Epiploic appendagitis

Epiploic appendages are small projections of adipose tissue that extend from the serosal surface of the bowel, primarily associated with the colon. These pedunculated fatty structures are arranged in two longitudinal rows along the anterior and posterior taenia coli on the external surface of the colon [116]. Epiploic appendages typically measure 1–2 cm in thickness and 0.5–5 cm in length [117].

Primary epiploic appendagitis is observed across a wide age range, from 12 years to 82 years, with the peak incidence occurring during the fifth decade. This condition demonstrates a higher prevalence among obese patients and females [118, 119]. Anatomically, primary epiploic appendagitis is most commonly localised to the sigmoid colon, followed by the caecum and ascending colon.

Epiploic appendages are also predisposed to torsion and subsequent ischaemic or haemorrhagic infarction, leading to frequent clinical presentation with acute abdominal pain.

On pelvic MRI, the affected epiploic appendage typically appears as a well-defined, oval or round lesion located adjacent to the colonic wall. Generally, it is surrounded by localised fluid adjacent to the colon and appears hyperintense on T1-WI, although slightly less intense than normal peritoneal fat. On suppressed T2-WI, the lesion demonstrates a marked loss of signal, confirming its fatty composition. Additional imaging features include evidence of a thin peripheral rim and perilesional inflammatory changes showing marked enhancement on contrast-enhanced T1-WI sequences. Furthermore, a central draining vein within the epiploic appendage can exhibit low SI on both T1- and T2-WI (Fig. 10) [120]. Epiploic appendagitis is a self-limiting condition, resolving spontaneously within 5–7 days under conservative management. Rare complications, including adhesions, bowel obstruction, intussusception, intraperitoneal loose bodies, peritonitis, or abscess formation, have been reported [121].

In the context of acute abdominal pain, the differential diagnosis includes diverticulitis, infarction of the omentum, appendicitis and, less commonly, mesenteric panniculitis or neoplasms of the omentum (both primary and metastatic).

#### Mesenchymal tumours

Mesenchymal tumours include a broad spectrum of benign and malignant lesions that can occur almost anywhere in the human body.

**Fig. 12:**
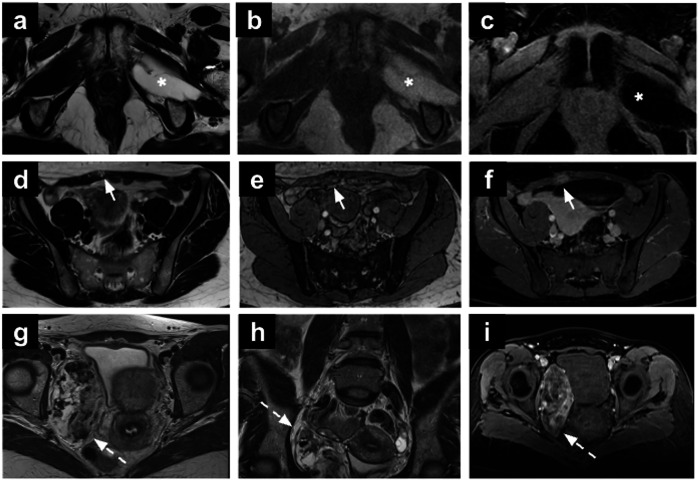
Lipoma (**a**–**c**). Vaginal cancer patient showing at the level of the left obturator externus, a large regular and homogeneous formation (white asterisks), appearing hyperintense on both T2-WI (**a**) and T1-WI (**b**) images and hypointense on fat-sat T1-WI (**c**), suggestive of intramuscular lipoma. Desmoid tumour (**d**–**f**). Axial T2-WI (**d**) and opposed-phase T1-WI (**e**) images from a 42-year-old woman undergoing MRI for adnexal mass characterisation showing a subtle heterogeneous signal mass within the right rectus abdominis muscle, better depicted as hypervascular on post-contrast axial T1-WI (**f**), pathologically confirmed as desmoid tumour. Aggressive angiomyxoma (**g**–**i**). Axial (**g**) and coronal (**h**) T2-WI show a hyperintense mass (dotted arrows) with swirled appearance and hypointense linear foci within the right hemipelvis, heterogeneously enhancing on post-contrast axial T1-WI (**i**), in keeping with aggressive angiomyxoma in a 57-year-old woman. WI, weighted images

Pelvis and abdominal wall tumours are overall rare and predominantly affect asymptomatic women. Tissue characterisation, such as the presence of fat, myxoid fibrous or vascular components, can narrow the differential diagnosis (Fig. 12).

Common mesenchymal tumours include lipoma, desmoid tumours and aggressive angiomyxoma [122, 123].

##### Lipoma

Lipomas are the most common fat-containing lesions, accounting for approximately 50% of benign soft tissue masses with an estimated prevalence of 2.1 per 100 individuals. There is no clear gender predilection or age distribution, as the age of presentation ranges from 25 years to 84 years [98, 124, 125]. Lipomas are typically located in the superficial soft tissue of the back and the extremities but may also be found deep to the superficial fascia [126].

On MRI, lipomas are well-defined lesions, showing homogeneous high SI on T1 and T2-WI, similar to subcutaneous fat, and low SI on fat-saturated images [98].

Features favouring benignity include superficial location (lesions in the subcutaneous tissue without fascia involvement), homogeneous fat signal, thin septa (up to 2 mm), persistence of vessels and intermingled muscle fibres within the lesion, a lobulated appearance, and a size of less than 5 cm for deep lesions or less than 10 cm for superficial lesions [98, 126, 127].

Lipomas are typically encapsulated, but intramuscular lipomas may lack clear borders and infiltrate muscle tissue [127, 128].

Features for malignancy include a deep location, lesion size > 10 cm, and the presence of a solid, non-fatty component [126]. If suspicious features are present, patients should be referred to a sarcoma centre; otherwise, follow-up or excisional biopsy are viable options [126].

##### Desmoid tumour

Desmoid tumours represent the most common abdominal wall lesion, accounting for approximately 30% of abdominal wall masses [122, 129]. These tumours predominantly arise in the anterior abdominal wall, often at sites of prior surgery or trauma. Despite their benign nature, desmoid tumours exhibit significant local invasiveness and a high recurrence rate.

On imaging, they typically appear as intramuscular solid lesions with either circumscribed or ill-defined margins and a characteristic band-like morphology. MRI findings vary depending on the tumour’s predominant component. Tumours with a myxoid-dominant composition exhibit low SI on T1-WI, intermediate-to-high SI on T2-WI, and vivid post-contrast enhancement. Conversely, lesions with a fibrous-dominant component demonstrate low SI on T2-WI and mild, delayed contrast enhancement [122, 123, 129]. In case of suspected desmoid tumour, confirmation with biopsy is necessary, and patients should be referred to experienced centres to ensure appropriate management, which may involve strict surveillance or surgery [130].

##### Aggressive angiomyxoma

Aggressive angiomyxomas are typically observed in the pelvis of middle-aged women, although rare cases originating in the abdominal wall have been reported [131, 132]. These benign, slow-growing lesions are highly infiltrative and often involve multiple compartments, leading to a significant risk of suboptimal surgical resection and high rates of local recurrence.

On MRI, they appear as laminated lesions showing high SI on T2-WI, due to the myxoid matrix, and enhancing avidly following contrast administration [133, 134]. Surgery represents the standard treatment. Therefore, precise delineation of the tumour’s anatomical extent and involvement of adjacent structures is critical for optimal surgical planning [123].

## Conclusions

Numerous incidental findings can be encountered in the assessment of the female pelvis MRI. As the gynaecological MRI protocols typically include multiple sequences acquired in various planes and with differing FOVs, a thorough evaluation of all images is essential to ensure that no findings are overlooked. Adopting a systematic approach that evaluates the anterior, posterior, lateral, and musculoskeletal compartments, as proposed, can reduce the risk of overlooked findings.

This structured framework also aids radiologists in characterising incidentally detected lesions, according to their anatomical site.

## Abbreviations

ADC: Apparent diffusion coefficient
BC: Bladder cancer
CN: Canal of Nuck
CRC: Colorectal cancer
DWI: Diffusion-weighted images
EMH: Extramedullary haematopoiesis
ESUR: European Society of Urogenital Radiology
FOV: Field-of-view
GIST: Gastrointestinal stromal tumour
IBD: Inflammatory bowel disease
IF: Insufficiency fracture
PCS: Pelvic congestion syndrome
SI: Signal intensity
STIR: Short tau inversion recovery
UC: Urachal cysts
UD: Urethral diverticulum
WI: Weighted images
